# Algorithm-Based Hearing and Speech Therapy Rehabilitation after Cochlear Implantation

**DOI:** 10.3390/brainsci12050580

**Published:** 2022-04-29

**Authors:** Theda Eichler, Wiebke Rötz, Christoph Kayser, Felix Bröhl, Michael Römer, Arne Henning Witteborg, Franz Kummert, Tobias Sandmeier, Christoph Schulte, Patricia Stolz, Katharina Meyer, Holger Sudhoff, Ingo Todt

**Affiliations:** 1 Department of Otolaryngology, Head and Neck Surgery, Medical School OWL, Campus Bielefeld Mitte, Bielefeld University, 33604 Bielefeld, Germany; wiebke.roetz@klinikumbielefeld.de (W.R.); holger.sudhoff@klinikumbielefeld.de (H.S.); ingo.todt@klinikumbielefeld.de (I.T.); 2 Faculty of Biology, Cognitive Neuroscience, Bielefeld University, 33615 Bielefeld, Germany; christoph.kayser@uni-bielefeld.de (C.K.); felix.broehl@uni-bielefeld.de (F.B.); 3 Faculty of Economics, Decision Analytics, Bielefeld University, 33615 Bielefeld, Germany; michael.roemer@uni-bielefeld.de (M.R.); henning.witteborg@uni-bielefeld.de (A.H.W.); 4 Research Institute for Cognition and Robotics, Bielefeld University, 33615 Bielefeld, Germany; franz@techfak.uni-bielefeld.de (F.K.); tsandmeier@techfak.uni-bielefeld.de (T.S.); cschulte@techfak.uni-bielefeld.de (C.S.); 5 Department of Design, University of Applied Science, 33619 Bielefeld, Germany; patricia.stolz@fh-bielefeld.de (P.S.); katharinam730@gmail.com (K.M.)

**Keywords:** cochlear implant, rehabilitation, artificial intelligence, hearing and speech therapy

## Abstract

Introduction: Due to the changes in the indication range for cochlear implants and the demographic development towards an aging society, more and more people are in receipt of cochlear implants. An implantation requires a close-meshed audiological and logopedic aftercare. Hearing therapy rehabilitation currently requires great personnel effort and is time consuming. Hearing and speech therapy rehabilitation can be supported by digital hearing training programs. However, the apps currently on the market are to a limited degree personalized and structured. Increasing digitalization makes it possible, especially in times of pandemics, to decouple hearing therapy treatment from everyday clinical practice. Material and Methods: For this purpose, an app is in development that provides hearing therapy tailored to the patient. The individual factors that influence hearing outcome are considered. Using intelligent algorithms, the app determines the selection of exercises, the level of difficulty and the speed at which the difficulty is increased. Results: The app works autonomously without being connected to local speech therapists. In addition, the app is able to analyze patient difficulties within the exercises and provides conclusions about the need for technical adjustments. Conclusions: The presented newly developed app represents a possibility to support, replace, expand and improve the classic outpatient hearing and speech therapy after CI implantation. The way the application works allows it to reach more people and provide a time- and cost-saving alternative to traditional therapy.

## 1. Introduction

Due to the changes in the indication range for cochlear implants, more and more people are given cochlear implants. Nevertheless, older people make up a large group of implantees. Demographic trends show that by 2050, one fifth of the entire human population will be over 65 years old [1,2]. The probability of hearing loss increases with age. For those affected, hearing loss means limited verbal communication [3]. For older hearing impaired people, hearing loss can lead to loneliness, depression, frustration and aggression [4,5]. In addition, the risk of falling increases [6]. All in all, hearing loss means a reduced quality of life. A study by the Bochum University Hospital clearly shows the effects of the extension of the indication criteria [7] and the ageing population towards an increasing number of cochlea implant (CI) implantations [8].

CI implantation alone does not lead to improved hearing performance. Implantation is usually followed by many years of therapy and technical fitting. The aim of CI fitting is to improve hearing and communication skills for increased social and professional participation, whereby hearing success is significantly influenced by audiological and speech therapy aftercare [9,10]. Structuration of the rehabilitation is variable and depends on maturational background. It can be divided into basic and follow-up therapy according to the local guidelines. It can be structured into a basic therapy lasting 3–5 days within the first six weeks after the implantation, while follow-up therapy can cover up to 40 days, within the first two years after implantation [7].

During therapy, the rehabilitation measures are individually adapted. In principle, a methodical approach is taken, in such a way that hearing therapy starts with an individual low level of difficulty. During the course of the therapy, the difficulty increases according to the hearing comprehension performance of the patient [11]. The therapy takes place on the level of perception, identification and differentiation, as well as the central auditory level of processing and understanding. In this context, auditory therapy can be supported, but currently not replaced, by digital auditory training programs [12].

Basic and follow-up therapy requires a high expenditure of time and personnel, which pose major challenges to the health system. What is needed is a significant reduction in costs with simultaneous improvement or standard-maintaining quality [13]. For this reason, initial approaches for digitally based aftercare have been developed for CI rehabilitation. The advancing digitalization and the accompanying increased demand for health apps among end users [14] requires a restructuring towards an autonomous and independent rehabilitation. Telemedical methods are a resource-efficient option, especially in sparsely populated areas. Völter et al. [15] recommend designing an individualized therapy concept in CI rehabilitation to efficiently care for the increasing number of CI patients due to demographic change and the extension of indication criteria [8]. Important factors of such a hearing training program are, besides the therapy success, the intuitive design, which encourages to continue the therapy. In order to ensure the success of the therapy, it is necessary to adapt it to the individual performance level of the patient [14]. In the English-speaking world, such rehabilitation concepts already exist [16,17], while in the German-speaking world the applications for auditory training are not individualized [15]. Völter et al. [18] developed a prototype of an application to establish a comprehensive app for hearing rehabilitation in German-speaking countries as well. The prototype is based on the concept of therapeutic guidance, which should enable outpatient logopedic care. The patients perform the exercises within the app independently, but they are still guided and supervised by a real speech therapist. The factor of supervision is a limiting point in terms of personal resources. The target group includes not only the young but even elderly patients with the willingness and ability to use mobile phone applications. Related to the aging society and the increasing group of older implantees, we tried to address this point by including elderly patients in the codesign of the app. Others have shown the usefulness of AI-based digital solutions for the elderly in their specific needs (e.g., detection of falls) [19].

The aim of our study was to create an app that allows independent and self-sufficient auditory training, without being dependent on the external supervision of a professional.

## 2. Materials and Methods

The development of the app was closely referred to the hearing therapies routinely performed at the Bielefeld Mitte University Hospital as described in [20]. Firstly, central structural points were determined. The app should fulfil the criteria of functionality, scientificity and data protection for medical apps. A high degree of content validity, as well as an intuitive and flexibly responsive operating concept, were also among the cornerstones of the development. The app not only takes into account the patient’s current level of performance at the time of the start of speech therapy rehabilitation, but also includes pre- and postoperative anamnesis as well as audiological diagnostics.

In an interdisciplinary team of hearing speech therapists, audiologists, CI surgeons, software developers, neuroscientists, economists and app designers, the concept of the app was first developed theoretically. Hearing training is based on the individual‘s starting point and independently adapts to the user’s progress. The initial situation is based on the factors influencing listening success as mentioned [21]. These include duration and time of deafness, age at implantation, cognitive ability, postoperative hearing threshold, and electrode location within the cochlea. Following Blamey et al. [22], even the influence of the fitting of the contralateral ear was evaluated. In addition, the intraoperative thresholds of the electrically evoked summation action potentials (eCAP) are included in the generation of the starting level of rehabilitation of the patients [23]. Table 1 shows the influential factors of the preoperative history, as well as intraoperative and postoperative diagnostics used in the app.

The primary goal of auditory training within the developed app is open speech understanding, i.e., understanding on a purely auditory basis without looking at the lip movement [24], and improved participation in everyday life. Therapy without a therapist seemed unthinkable just a few years ago. The COVID-19 pandemic highlighted the relevance of a low-contact form of therapy, and the increasing digitalization additionally pushed it forward. That is the reason why the app is designed to be therapist-less and to function independently of outpatient rehabilitation measures.

Through permanent feedback to the user and motivational concepts, such as varied exercises, and a simple but appealing design, created by app designers of the University of Applied Science Bielefeld, the app should encourage intensive use [25,26]. It is particularly important to ensure user-friendliness and orientation. Intrinsic motivation must be addressed sufficiently to evoke active engagement in the user and to achieve the therapeutic goal [27].

## 3. Results

### 3.1. Structure of the App

The app currently contains 1400 words, 650 sentences, 230 phrases, 22 texts, 26 syllables and 25 dialogs. In addition, 420 minimal pairs are stored in the app. The speech material was recorded by a female speaker. It was decided to have a female speaker due to the lack of experiences with speech therapies performed by male or gender neutral artificial voices. A female voice is therefore more in line with the therapy reality of CI patients [28] and known to generate reliable results. It might be a matter of debate to open this approach in the future [29]. In addition to the speech material, there are also 22 everyday sounds and tones, as well as 40 pieces of music and melodies, and 25 different voices (male/female/adult/children).

In total, the app contains 89 exercises of different levels of difficulty (very easy, easy, medium, and difficult) from eleven categories (sounds, pitches, single sounds, syllables, words, phrases, sentences, texts, free speech, voices, and music). Depending on the exercise, the app can provide up to 20 different levels of support (aids) with different strengths (light, medium, and strong). In addition to the training units in silence, exercises in noise are implemented. To ensure realism, the recordings for exercises in noise were made in babble noise presented via headphones. Spoken speech in babble noise produces the greatest changes in loudness, speech rate, articulation, and pitch (Lombard speech) [30].

The app is divided into two parts: an expert view and a user view. The data in the expert interface can be viewed by the patients but cannot be changed. This is where the anamnestic and diagnostic factors are stored. The user interface is intended for everyday use of the app. Within the user interface, the patient can not only perform the exercises, but also independently check his success through implemented speech intelligibility tests. The speech intelligibility tests are not performed in the open field, but, like the entire training, via the direct connection to the CI. In addition, user statistics, such as duration of use or progress analysis, are provided to give the patient the possibility to check his listening success. Such an independent review and tracking of progress additionally contributes to the patient’s motivation.

### 3.2. Initialisation and Use

The app has to be initialized after the third fitting by the audiologist responsible. Primarily, the audiologist performs a cognitive assessment using the DemTec [31,32] and the MoCa [33]. The parameters listed in Table 1 are entered via the expert interface. Based on the factors of the anamnesis described in Table 1, as well as the intraoperative and postoperative diagnostics, an individual starting level is determined. An initial forecast is then given for the duration until open speech understanding is achieved. Based on this forecast, the increase in exercise difficulty is either slowed down, accelerated or kept stable.

Exercises can be performed via the user interface. Before each exercise session, the patient is instructed to perform another, highly abbreviated attention test to determine the duration and intensity of the respective exercise session. This ensures that the patient is not overburdened by a high level of demand and that the training is adapted to the patient’s daily form. In addition, the volume should be set at a level that is easy for the patient to hear. After each technical setting, the audiologist stores the programs stored in the processor in the app. In this way, the app can refer to the stored louder setting if volume is missing. This also enables the patient to try out the stored programs and the various directional characteristics according to instructions of the app.

The default value for the duration of a standard exercise session is 25 min. This time should be kept at least to avoid habituation and fatigue effects. Such a practice session should be performed at least once a week to achieve good listening comprehension. The practice session only includes the pure practice time, without the volume adjustment and the attention tests. In addition to the exercise session, the patient has the opportunity to complete other shorter exercises. These do not count towards the progress rating and should only serve to train the patient.

### 3.3. Adaptivity

An important element of the app is the adaptivity of the difficulty level of the exercises. Adaptivity occurs at three levels: within the exercise via the use of aids, in the increase in complexity of the language material across exercises, and in the listening environment.

Within the exercise, there is an automatic and dynamic adaptation of the level of difficulty by means of the aids. The selection of the aids given the state of the patient is based on a parametric policy. An initial parameterization of this policy was specified by the therapists from Bielefeld University. Once more and more data are collected in the use of the app, the policy will be improved using actor-critic machine learning approaches. In addition, these approaches will be used to adapt the aid selection policy to each individual patient during the training process. In this context, note that the overall goal is not to provide the aid that most likely leads the user to the correct answer, but to reduce the use of aids in general. The use and adaptation of the aids only offer the possibility of a careful habituation to the level of difficulty. The language material is adapted by means of the aids by varying the pronunciation or length of the material. In addition to changing the language material, visual aids can also be presented, such as showing the initial letter. A maximum of seven helps per item (word/sentence/phrase, etc.) are offered. This always includes the simple repetition of the item and two aids of each of the three different strengths. If the item still cannot be entered, it is considered as not understood.

**Figure 1:**
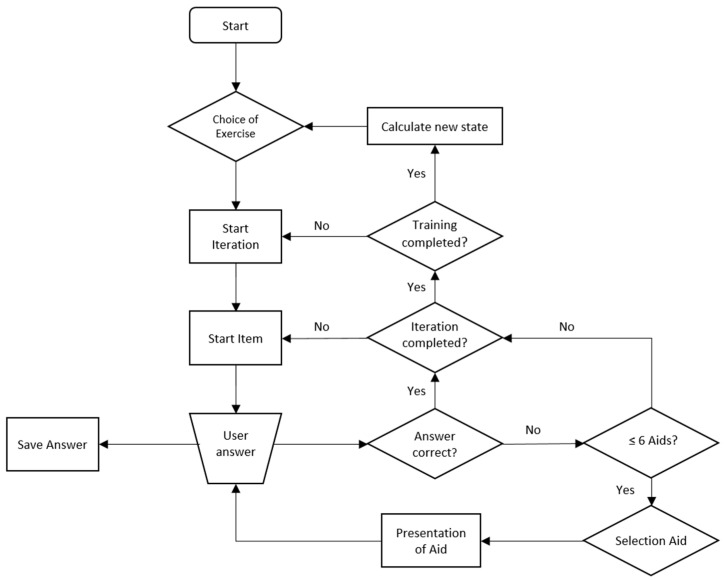
Program procedure plan for an adaptivity algorithm within the exercise.

The app continuously records the number and type of errors, as well as the aids used. The lower the number and strength of help needed, the more likely the complexity of the language material will increase (e.g., sentences instead of phrases). If there is stagnation within a difficulty level, despite diverse helps within an exercise, a decrease in the complexity of the language material is also possible. As for the selection of aids, the policies for selecting exercises and for increasing and decreasing difficulty levels are improved in a data-driven fashion using machine learning approaches. Figure 1 outlines the simplified program flow of an exercise. An exercise consists of several iterations (blocks). The current complexity of the language material is called state.

In addition to adjusting the difficulty level within the exercises and the speech material, the listening situation is also changed when a high level of speech comprehension is reached. The speech material is overlaid with various noises (e.g., babble noise). The listening situation can be influenced by automatically adjusting the signal-to-noise ratio (SNR).

### 3.4. Problem Analysis

The goal of follow-up and basic therapy is to ensure optimal benefit for the patient. To achieve this, the dovetailing of fitting and hearing therapy is necessary [7]. Accordingly, a self-sufficient app should also offer fitting control. Within an outpatient therapy session, the therapists evaluate difficulties in sound identification and differentiation as well as the correct adjustment of the overall volume [34].

By storing the word material in phonetic transcriptions (according to the online dictionary wikitionary.com [35]), the app is able to perform a background analysis on which sounds are problematic, based on the input errors from the exercises. These are verified within the single-sound exercises for inconsistent and consistent sound confusions and omissions. Frequency-specific problems can additionally be identified within the noise exercises. In this way, it is possible to evaluate which frequency ranges are underrepresented. The patient is then encouraged to see the appropriate audiologist to pass on the information to the person making the adjustment (see Table 2).

The overall volume level is checked by the app at the beginning of each exercise session. If the maximum volume level is not sufficient, the patient will be prompted to visit the audiologist responsible for a volume adjustment.

### 3.5. Benefits of the Algorithm

Due to a potentially large group of subjects and an upcoming continuous use of the app, the machine learning/artificial intelligence (ML/AI)-based algorithms will be able to implicitly capture correlations leading to an improved sequencing of exercises and selection of aids. During use, all data is stored. In addition to storing the error analyses and the aids used, the progress and regress are also noted. These are linked to the initially stored data of the medical history and pre- as well as postoperative diagnostics. The algorithms identify patterns, e.g., the frequency of errors in certain exercises, the best-performing aids or the speed of progress. Thus, the algorithms are continuously trained and are able to apply a consequence for specific patient groups. This means that as the app evolves, patients are presented with more targeted aids and/or experience a slower or faster increase in the difficulty of the exercises. In this way, the individual processes can be personalized even more, and the success of the therapy can be increased. Such a link is also useful for outpatient therapy. For patients who are unable to use the app, the patterns identified by the app in other patients can lead to an adjustment of the therapy and provide guidelines for the therapy of specific patient groups.

### 3.6. State of the App

**Figure 2:**
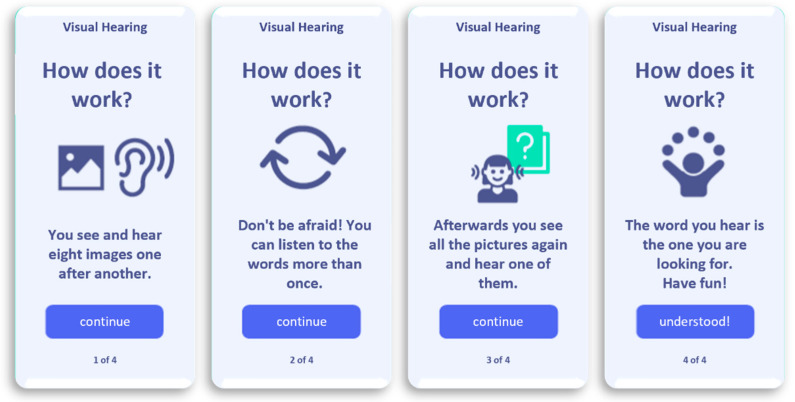
Exemplary illustration of the explanation for an easy word exercise.

**Figure 3:**
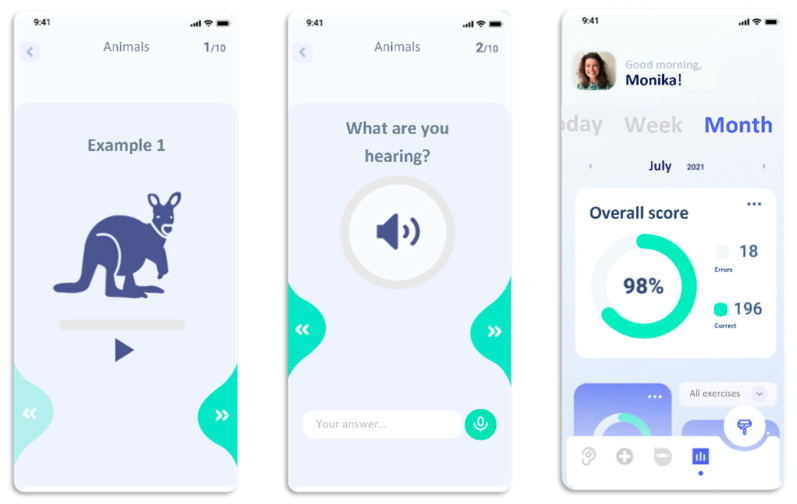
Exemplary illustration of a word exercise (left and middle) and of a performance overview (right).

The current third version of the app is tested by including experienced cochlea implantees of different ages to improve the usability of the software. This co-design called element allows a user-friendly improvement of the developed version (see exemplary illustrations in Figure 2 and Figure 3). The period of wireframing of the app was performed by an interdisciplinary work between designer, software developers, speech therapists, audiologists and cochlea implantees. After including data security elements, a pilot study will be started for a period of 3 to 6 months; subsequently, following a possible successful evaluation of the results, a clinical evaluation after about one year is designed and underway.

## 4. Discussion

The potential with eHealth apps is promising. In addition to inclusion of underserved populations such as rural populations or those with chronic illnesses, increasing treatment adherence is also a critical factor [36]. A general problem with implementing AI-based treatment interventions is the acceptance of the lack of human interaction. According to [37], a large proportion of patients reject therapeutic interventions without therapist contact. Irvine et al. [38] found that telephone-based therapy and face-to-face therapy had no difference in effect. However, the COVID-19 pandemic is making telemedicine and the proliferation of eHealth apps increasingly important [39,40]. The pandemic is crucial for a higher acceptance of digitalization in everyday life. Therapy through an app is accordingly more accepted today, especially among the younger generation with an affinity for smartphones [41]. In this context, flexibility in terms of time and health security are crucial for patients.

The developed app presented here for hearing rehabilitation after cochlear implantation is intended to possibly replace outpatient hearing and speech therapy. Despite the orientation towards the outpatient rehabilitation measures at our hospital, there are some differences in the implementation of the therapeutic measures in a direct comparison. While a therapist can address individual needs right from the start, the app is initially individualized to a limited extent. However, the app is capable of learning, thanks to the stored AI algorithms. With greater intensity of use, the app should be able to tailor the therapy to the patient and thus individualize it, and precisely determine and counteract errors in the setting. A disadvantage of the missing therapist contact is the prolonged way to improve the technical setting of the speech processor by the audiologist.

Initial approaches to audiologist-free intelligent technical fitting have already been developed (Fitting to Outcomes eXperts (FOX™), Otoconsult NV, Antwerp, Belgium). Waltzman et al. [42] compared the FOX™ artificial intelligence technical settings with those of traditional audiologists. The results suggest that the technical settings of artificial intelligence can be equivalent to the traditional method.

Through ML/AI algorithms, the app will be able to adapt the hearing rehabilitation to the patient’s individual strengths or weaknesses in order to achieve maximum therapeutic success. The app consists of 89 different exercises of varying difficulty from eleven different categories such as sentences, phrases or words.

Individualization of the app is achieved through adaptive adjustment of the difficulty level. By dynamically adjusting the level of difficulty, the app ensures that patients are able to solve the tasks they are given. On the other hand, it ensures that patients do not lose interest in the therapy due to unsolvable tasks and that the success of the therapy is not endangered.

A disadvantage of using an app instead of outpatient therapy is the lack of control over the patients’ duration and intensity of use [43]. To overcome this problem, the app itself determines the intensity and duration of each exercise session based on data obtained from the user (daily form, last exercise difficulty). The app reminds the patients to perform the exercise session on the days specified by the patients in advance. Thus, a lack of use may indicate a lack of intrinsic motivation [27]. Accordingly, relevant for the consistent use of the app is the right intrinsic, as well as extrinsic motivation through an appropriate level of difficulty, goal-oriented instructions, a high degree of self-determination, and an appealing, intuitive design. This is consistent with the tenets of self-determination theory [44].

The app is potentially suitable for any post-lingually deaf, German-speaking adult or adolescent. In the case of a prevailing rejection of digital media, an existing mental or visual impairment, as well as rheumatic diseases, the use of the app is difficult to impossible. Especially for unilaterally deafened people, therapy via an app can be beneficial. Due to the direct connection to the smartphone/tablet, there is no risk of overhearing on the normal-hearing side. However, bilateral or bimodal patients can also benefit from the flexibility and practicality of a hearing rehabilitation app. In order to train hearing not only with the help of one voice, the app will be expanded in the future to include a male voice. This way, the entire frequency spectrum of speech can be covered.

In addition to the benefits of a hearing rehabilitation app for patients, science should also benefit from such an app. With computer-based hearing training, it might be possible to standardize hearing therapy and evaluate it scientifically in further steps. By storing the therapy and medical history data, the app potentially should be able to recognize correlations through self-learning algorithms and to increasingly specify and adapt the training to the patients in the future. It can also enable the development of alternative outpatient rehabilitation approaches that are adapted to the patient’s medical history from the outset.

For the clinics, telemedicine means an economic advantage. As a telemedical measure, hearing and speech therapies with an online face-to-face therapist are carried out and positively accepted by many patients at our hospital. An app to replace the therapist would be the logical next step. To prove the acceptance and evaluate the benefits of such an app, a study at Bielefeld University Hospital is planned. A group of users will use the app as therapists from the beginning, while a comparison group will use the traditional way of an outpatient therapy, whether in person or online. After three months, there will be a short-term evaluation to compare the results. In case of significant differences, the study will be stopped. Furthermore, the data will be compared with those of the previous years.

## 5. Conclusions

The presented newly developed app represents a possibility to support, replace, expand and improve the classic outpatient hearing and speech therapy after CI implantation. In addition to the potentially improved therapy success of the patients, the app might also offer a number of advantages for the hospital, as well as for the payers, since with the help of the app, significantly more patients could be treated at the same time, with significantly lower personnel costs. With the features of the app described here, it represents a potential opportunity to make therapy in the field of hearing rehabilitation significantly more efficient and to provide patients with the option of flexible and individual therapy.

## Figures and Tables

**Table 1:** Factors influencing the starting level.

Anamnestic Factors	Intraoperative Diagnostics	Postoperative Diagnostics
Previous duration of hearing loss/deafness	eCAPs	Speech processor and connection options
Cause of deafness	Impedances	Wearing times
Previous treatment of hearing loss	Abnormalities (e.g., ossifications)	Hearing and speech tests (from third fitting)
Residual hearing in implanted ear	Electrode position, insertion depth	Residual hearing (possibility of EAS system)
Sound/sign language socialization	Manufacturer of the implant	Neurocognitive abilities
Information on opposite side (normal hearing, hearing aid, surditas)		
Illiteracy		
Mother tongue		

Note: eCAPS (electrically evoked compound potential), intraoperative measurement of the auditory nerve in CI patients; EAS (electric-acoustic stimulation), possibility of using the residual hearing.

**Table 2:** Problems of sound differentiation and omission.

Problem	Feedback to the Audiologist
Not heard at all	Frequency range too quiet
	Other frequency range too dominant
Consistent confusion	Corresponding frequency range too quiet
	Corresponding other frequency range too dominant
Inconsistent confusion	Can usually be influenced by practice
	insufficient habituation to the existing setting

## Data Availability

Not applicable.
